# Supplementary material for: Newly Initiated Statin Treatment Is Associated with Decreased Plasma Coenzyme Q10 Level After Acute ST-Elevation Myocardial Infarction
Source: Int J Mol Sci. 2024 Dec 26;26(1):106. doi: 10.3390/ijms26010106 (PMC11720258; doi:10.3390/ijms26010106)
Supplement: Supplementary file 1 [file ijms-26-00106-s001.zip › Supplementary Table 1.pdf]

Supplementary table 1. The course of lipid-lowering treatment (LLT) regimens during the study. The grey areas represent the timepoints. where CoQ10 samplings were performed.

Lipid-lowering treatment (LLT)	Baseline LLT at hospital admission		LLT at hospital discharge		LLT at 3-month follow-up		LLT after 3-month follow-up	
	Number	%	Number	%	Number	%	Number	%
High-intensity statin therapy	0	0.0	65	97.0	62	92.5	63	94.0
• Atorvastatin 40 mg	0	0.0	3	4.5	3	4.5	5	7.5
• Atorvastatin 80 mg	0	0.0	0	0.0	0	0.0	0	0.0
• Rosuvastatin 20 mg	0	0.0	62	92.5	56	83.6	51	76.1
• Rosuvastatin 30 mg	0	0.0	0	0.0	0	0.0	1	1.5
• Rosuvastatin 40 mg	0	0.0	0	0.0	3	4.5	6	9.0
Moderate-intensity statin therapy	0	0.0	2	3.0	4	6.0	4	6.0
• Atorvastatin 10 mg	0	0.0	0	0.0	1	1.5	2	3.0
• Atorvastatin 20 mg	0	0.0	2	3.0	3	4.5	1	1.5
• Rosuvastatin 10 mg	0	0.0	0	0.0	0	0.0	1	1.5
Ezetimibe therapy	0	0.0	1	1.5	2	3.0	19	28.4
• Monotherapy	0	0.0	0	0.0	0	0.0	0	0.0
• Combination with statin	0	0.0	1	1.5	2	3.0	19	28.4
No lipid-lowering therapy	67	100.0	0	0.0	1	1.5	0	0.0
