# Supplementary material for: Newly Initiated Statin Treatment Is Associated with Decreased Plasma Coenzyme Q10 Level After Acute ST-Elevation Myocardial Infarction
Source: Int J Mol Sci. 2024 Dec 26;26(1):106. doi: 10.3390/ijms26010106 (PMC11720258; doi:10.3390/ijms26010106)
Supplement: Supplementary file 1 [file ijms-26-00106-s001.zip › Supplementary Table 2.pdf]

Supplementary Table 2. Determination of predictor(s) of CoQ10 as a dependent variable using backward stepwise multiple regression analysis in STEMI patients after 3-month statin therapy

Variable	$\beta_{standardized}$	P-value
Age (yrs)	0.06	0.66
BMI (kg/m^2)	0.14	0.32
Total cholesterol (mmol/L)	**0.34**	**0.01**
LDL-C (mmol/L)	-0.47	0.26
Oxidized LDL (U/L)	-0.04	0.92
Apo B100 (g/L)	-0.19	0.77
HDL-C (mmol/L)	0.23	0.27
Apo AI (g/L)	-0.12	0.44

Abbreviations: Apo AI, apolipoprotein AI; Apo B100, apolipoprotein B100; BMI, body mass index; CoQ10, Coenzyme Q10; HDL-C, high-density lipoprotein cholesterol; LDL, low-density lipoprotein; LDL-C, low-density lipoprotein cholesterol.
