# Supplementary figures and images for: Newly Initiated Statin Treatment Is Associated with Decreased Plasma Coenzyme Q10 Level After Acute ST-Elevation Myocardial Infarction
Source: Int J Mol Sci. 2024 Dec 26;26(1):106. doi: 10.3390/ijms26010106 (PMC11720258; doi:10.3390/ijms26010106)

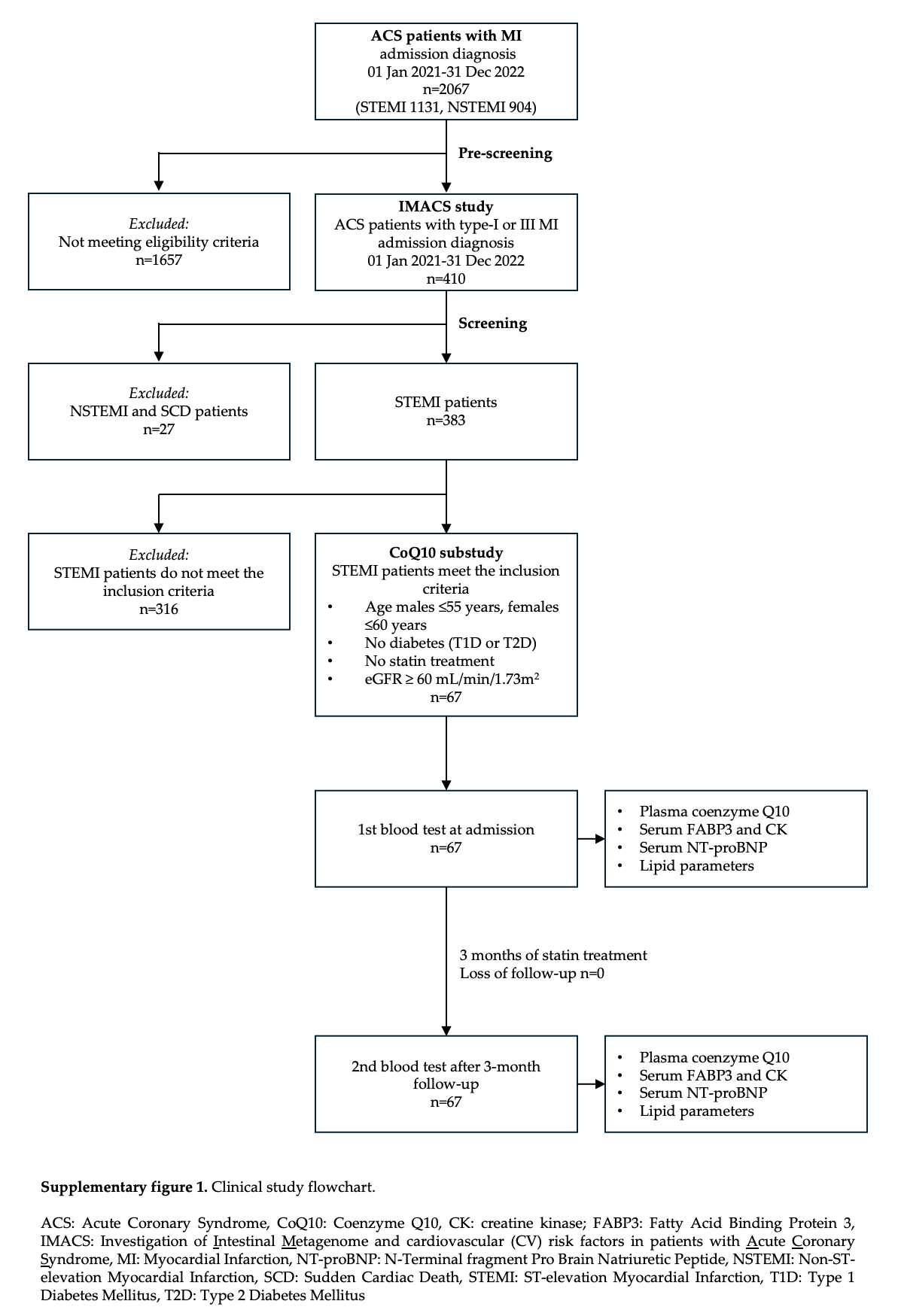

Supplement: Supplementary file 1 [file ijms-26-00106-s001.zip › Supplementary Figure 1.tiff]
